# Supplementary material for: Identification of oxidative stress-responsive genes in recurrent miscarriage and their role in disease pathogenesis
Source: PLoS One. 2025 Dec 2;20(12):e0337362. doi: 10.1371/journal.pone.0337362 (PMC12671832; doi:10.1371/journal.pone.0337362)
Supplement: S1 Table — (DOCX) [file pone.0337362.s001.docx]

## Table S1. List of OSRGs

| OSRGs | | | | |
| --- | --- | --- | --- | --- |
| *KIT* | *ATF4* | *CALR* | *IL4* | *SECISBP2* |
| *NDUFA13* | *GAPDH* | *MIF* | *ST3GAL4* | *CCNB1* |
| *MAPK13* | *CDKN2A* | *GFAP* | *NR1I2* | *CSF2* |
| *TNFSF4* | *CCL2* | *ETV5* | *INPP5D* | *CYP2B6* |
| *OXTR* | *MSRB2* | *ARL6IP5* | *CTSS* | *PSMC3* |
| *DHFR* | *BCL2L1* | *CDH13* | *IDE* | *PSMA4* |
| *ACACA* | *NOS1* | *VIM* | *GADD45A* | *GADD45GIP1* |
| *GPX8* | *SIRT3* | *PSIP1* | *ATG4B* | *ANGPTL7* |
| *TAZ* | *SRXN1* | *RORA* | *STK3* | *CSF1* |
| *MAPK12* | *CYP1A1* | *OXA1L* | *EIF4EBP1* | *PGF* |
| *YBX1* | *HIF1A* | *PLAT* | *TNFSF11* | *SEMA4A* |
| *SFXN4* | *CYP2E1* | *PGD* | *CSNK1D* | *CAPN1* |
| *SLC4A1* | *SRC* | *MAP2K2* | *NDUFS3* | *MMP7* |
| *HPSE* | *PPARGC1A* | *YWHAE* | *HSPA1L* | *MSR1* |
| *RPTOR* | *SIRT2* | *CASP2* | *ELN* | *HES1* |
| *MTA1* | *ATF2* | *MCTP1* | *HSD17B10* | *TRPC1* |
| *CD274* | *GGT1* | *NCF2* | *NME5* | *SGCB* |
| *ENDOG* | *ANXA5* | *MBL2* | *IPCEF1* | *HYOU1* |
| *BMP4* | *CYGB* | *DNMT1* | *CBR1* | *XPA* |
| *MTTP* | *JUNB* | *EGF* | *SP3* | *BID* |
| *TCF7L2* | *ATM* | *SPP1* | *COL2A1* | *PSMA2* |
| *TLR6* | *VEGFA* | *FLT1* | *EXOC6* | *UHRF1* |
| *VDR* | *GPX4* | *ETFDH* | *HSPA2* | *HSPB6* |
| *NFU1* | *MGST1* | *NLRX1* | *NDUFA8* | *FAM3A* |
| *GADD45G* | *APOE* | *PRKCQ* | *NDUFS6* | *CAPN9* |
| *FMO4* | *PPARG* | *UGT1A6* | *NDUFV2* | *OSCAR* |
| *AREG* | *TMEM161A* | *ELAVL1* | *HSPA12A* | *RBPMS* |
| *HK1* | *TF* | *CAMK2D* | *NDUFA7* | *FLT4* |
| *PIK3R2* | *PTGS2* | *TRIM26* | *UBE2I* | *L2HGDH* |
| *ATG5* | *CREB1* | *SLC23A2* | *F3* | *SLC1A5* |
| *SIRT6* | *CASP9* | *SLC3A2* | *PCK1* | *LPA* |
| *JAZF1* | *NUDT1* | *HSF1* | *BTK* | *FUT8* |
| *FADD* | *FOXO4* | *ABCB10* | *VHL* | *EIF4ENIF1* |
| *BCL2A1* | *PRKCB* | *WDR26* | *AIF1* | *SMPD3* |
| *IL11* | *GLRX* | *TREM2* | *HTR2A* | *NBN* |
| *MAP3K11* | *LMNA* | *POMC* | *DEGS1* | *CRH* |
| *CYP4F2* | *DHCR24* | *PLA2G3* | *COQ2* | *PTGER2* |
| *ASPA* | *MAPKAPK2* | *TLR2* | *ENO2* | *PCCB* |
| *ROCK1* | *FXN* | *FBXW11* | *TRIM63* | *PDCD4* |
| *NEDD8* | *PLA2G7* | *AOC3* | *PIM1* | *IPO7* |
| *GAA* | *NOL3* | *LGALS3* | *PTX3* | *IL6ST* |
| *PEPD* | *AGTR1* | *BCHE* | *NONO* | *SOX9* |
| *CAPN2* | *CAV1* | *KDR* | *CTSH* | *C1QTNF3* |
| *PTPRC* | *NFE2L1* | *ERCC8* | *CTSZ* | *PKP3* |
| *CYP20A1* | *PRKCA* | *LOXL4* | *NDUFB9* | *NR1H4* |
| *MKI67* | *MMP2* | *HSP90AB1* | *DNAJB2* | *HTRA1* |
| *SORL1* | *PPIA* | *NAGLU* | *NDUFB10* | *RGN* |
| *SORD* | *AGER* | *S100B* | *NDUFA10* | *APOA4* |
| *MUC5AC* | *DDIT3* | *NDUFS2* | *NDUFB7* | *SREBF2* |
| *ALDH3A2* | *GLRX2* | *ATF3* | *NDUFV3* | *LYAR* |
| *CHCHD2* | *TXNRD2* | *PPP3CA* | *NDUFA3* | *MAP3K2* |
| *PLD1* | *MAP2K4* | *SFN* | *HSPA12B* | *POLI* |
| *C5* | *OXR1* | *SLC25A4* | *NDUFB5* | *MAP1LC3B* |
| *TNFRSF11B* | *NLRP3* | *BNIP3* | *PPIG* | *PABPC1* |
| *TNFRSF10A* | *HMGB1* | *SLC6A3* | *FOXL2* | *PIAS4* |
| *FABP1* | *JUND* | *GSTO2* | *PRKDC* | *GLRX3* |
| *PFKM* | *HSPA5* | *IKBKB* | *ABCC2* | *ADRB1* |
| *IL6R* | *MDM2* | *FASLG* | *ERCC3* | *SLC4A2* |
| *SUMO1* | *ACE* | *LPO* | *NQO2* | *AFG3L2* |
| *TEK* | *RPS6KA5* | *SELP* | *SCARB1* | *CYB5R3* |
| *SLC7A1* | *TERT* | *IREB2* | *HDAC4* | *FASN* |
| *FIG4* | *NR2C2* | *EPAS1* | *CD14* | *STAU2* |
| *GLT8D1* | *MAP2K6* | *HDAC6* | *ACADS* | *CALM1* |
| *LBR* | *ICAM1* | *SLC25A24* | *ACOX1* | *ADRB2* |
| *CXCL16* | *LEP* | *ACP1* | *PNPT1* | *GPI* |
| *OSM* | *GSS* | *NOX5* | *RBX1* | *IL17RA* |
| *GRIN1* | *IL1B* | *ACE2* | *SLC19A1* | *SLC6A2* |
| *DYRK1A* | *VCAM1* | *NGF* | *IDH2* | *C1QBP* |
| *DNM2* | *CDKN1A* | *ATP13A2* | *PLAUR* | *GRP* |
| *TACO1* | *EDN1* | *ATP2A2* | *TGFA* | *HTATIP2* |
| *GIGYF2* | *GSTM3* | *NDUFA6* | *MAP3K7* | *PSMD10* |
| *C5AR1* | *MAPK9* | *CASP1* | *CCL20* | *GLP1R* |
| *ALDH3B1* | *KRAS* | *IL2* | *MYB* | *PDK2* |
| *CCR7* | *BCL2L11* | *ELANE* | *COL1A1* | *PIWIL4* |
| *MUC1* | *STUB1* | *TRAP1* | *PPARD* | *BDH2* |
| *CANX* | *PRKD2* | *ALDH9A1* | *CST3* | *NCOA7* |
| *ADH1A* | *BACH1* | *IL1A* | *JAK1* | *TGFBR1* |
| *FRZB* | *STK24* | *SLC2A4* | *ERBB2* | *TNC* |
| *EEF2* | *SESN2* | *GFER* | *PIK3C2A* | *RHD* |
| *ZFAND1* | *MAPT* | *MT1X* | *AGAP3* | *CTH* |
| *UBQLN4* | *SERPINE1* | *IGF1R* | *RUNX3* | *SLC12A2* |
| *TNIP1* | *IL18* | *CCND1* | *UBQLN1* | *MYD88* |
| *SCN4B* | *NRF1* | *SETD2* | *PTMA* | *MARK2* |
| *IL16* | *GCLM* | *MT2A* | *RAD50* | *ICMT* |
| *ARNT* | *TNFAIP8L1* | *YWHAB* | *TYMP* | *MT1F* |
| *CYP19A1* | *NGB* | *EP300* | *NUP153* | *RYR1* |
| *SDC1* | *MAOA* | *NEIL2* | *MEF2A* | *DRD1* |
| *CACNA2D1* | *CTNNB1* | *HGF* | *NR4A1* | *FGFR1* |
| *AQP1* | *BAD* | *CDKN1B* | *MAVS* | *POLR2A* |
| *CDC25C* | *LRRK2* | *XRCC5* | *LAMP2* | *MTRR* |
| *ACTN4* | *PSEN1* | *CD40LG* | *WNT1* | *SCO2* |
| *BSG* | *RAC1* | *UCP1* | *PMAIP1* | *PTP4A1* |
| *LYRM4* | *STAT3* | *XIAP* | *HRAS* | *FABP2* |
| *KL* | *TRAF6* | *IGFBP7* | *SLC9A1* | *NUP88* |
| *ANXA11* | *SCARA3* | *KRIT1* | *CD163* | *COG2* |
| *AVP* | *CASP8* | *AMBP* | *RUNX2* | *TP53I3* |
| *ABCC3* | *PIK3CG* | *PAGE4* | *DDAH2* | *ROCK2* |
| *MRAP* | *CAMK2G* | *HSP90B1* | *PYROXD1* | *CYP2D6* |
| *FANCD2* | *PPIF* | *TRPA1* | *NEIL3* | *IGFBP1* |
| *FIS1* | *TLR4* | *C19orf12* | *PTPN11* | *E2F3* |
| *PGAM5* | *XRCC1* | *ADA* | *S100A8* | *PDGFRB* |
| *CAMKK2* | *AKR1B1* | *HDAC3* | *COQ9* | *ANGPT1* |
| *VIPR1* | *MTOR* | *SUV39H1* | *PGR* | *ATP6AP1* |
| *ADRB3* | *MAPKAPK3* | *SMAD2* | *SLC25A5* | *SLC25A13* |
| *RTN4* | *RAF1* | *TGM2* | *CALCA* | *RPS27* |
| *FAAH* | *NDUFS4* | *ENG* | *POU2F1* | *DGKZ* |
| *GLA* | *MAPK11* | *XPC* | *ALDH3A1* | *TNPO1* |
| *PDE4A* | *BRF2* | *TGFB2* | *EGLN2* | *IL37* |
| *CX3CR1* | *MMP9* | *STK39* | *NFIC* | *ELF5* |
| *NOD2* | *DAXX* | *TTR* | *NME1* | *BRD4* |
| *SFTPB* | *FOSB* | *YWHAH* | *MEFV* | *HSPA14* |
| *LANCL1* | *PRKAA1* | *PRKAB1* | *GLUD1* | *PCCA* |
| *CD38* | *RPS27A* | *CD44* | *AKT2* | *CD80* |
| *FECH* | *SESN1* | *ALOX12* | *EEF1D* | *TRIM27* |
| *TPPP3* | *XBP1* | *PTK2B* | *IL1RN* | *PSMC6* |
| *IL1RAPL2* | *MAP2K7* | *UCHL1* | *CNR1* | *RHOD* |
| *TP53INP1* | *CYP1B1* | *CLEC4A* | *LDLR* | *PAPPA* |
| *ENC1* | *DUSP1* | *ENO1* | *EZR* | *ACADVL* |
| *PEX5* | *BAK1* | *CXCL1* | *CDK1* | *CKB* |
| *IKBKG* | *HMOX2* | *FOXP2* | *PXN* | *ATP8A1* |
| *FYN* | *EGFR* | *PRMT1* | *PNKP* | *TALDO1* |
| *ABCG2* | *MTHFR* | *FGF2* | *KRT18* | *CAPN6* |
| *UCN2* | *VWF* | *G3BP1* | *CRYAA* | *CAPN7* |
| *CD46* | *ABL2* | *GABPA* | *APOA5* | *KIF12* |
| *KIAA0319L* | *BRCA1* | *POLB* | *DKK1* | *HSD17B4* |
| *CXCL2* | *EIF2S1* | *TPM1* | *SOAT1* | *PPP2R2A* |
| *GPX5* | *OLR1* | *EPHA3* | *NCL* | *NUP214* |
| *CD28* | *HP* | *ARAF* | *MAFG* | *GH1* |
| *YAP1* | *HFE* | *XRCC6* | *TRPM7* | *HMGA2* |
| *GADD45B* | *SLC1A1* | *DNAH8* | *XPO1* | *GRK4* |
| *EIF2AK4* | *AGT* | *IL3* | *CYLD* | *HOMER1* |
| *NEK1* | *DAPK1* | *ROMO1* | *PXDN* | *ZFHX3* |
| *HDAC9* | *STK4* | *NR3C2* | *ANKRD2* | *PCBP2* |
| *ACAD8* | *HSP90AA1* | *FNDC5* | *SLC22A4* | *FGFR2* |
| *ADAM10* | *MAP2K1* | *MAFF* | *UBIAD1* | *OPA1* |
| *RAG2* | *VKORC1L1* | *MAPK7* | *CASP4* | *TIMP2* |
| *C3* | *ADNP2* | *SIN3A* | *OCLN* | *CAPN3* |
| *HRH2* | *CDK4* | *FBXW7* | *TRPC3* | *EED* |
| *VEGFC* | *SLC17A5* | *ALDH7A1* | *TKT* | *TNFRSF10B* |
| *SLC8A1* | *FTL* | *KLHDC10* | *TTK* | *ACADM* |
| *MRPS34* | *EPO* | *RIPK1* | *DCT* | *RIPK3* |
| *IL12B* | *PTEN* | *PDE5A* | *ATG7* | *DPYSL2* |
| *CIITA* | *IL10* | *NDUFS1* | *SMPD2* | *SAT1* |
| *SIAH1* | *FAS* | *GLUL* | *FXYD1* | *MAP2* |
| *SET* | *AIFM1* | *REN* | *LMNB1* | *DBH* |
| *MMD* | *CLU* | *NNT* | *B2M* | *SCNN1A* |
| *ITGA2* | *ALAD* | *MTFR1* | *BAG3* | *ABCG1* |
| *TLR5* | *SENP3* | *LCN2* | *TRIM21* | *STEAP4* |
| *PHYH* | *ALDH2* | *IDH1* | *OPTN* | *KHSRP* |
| *HSPG2* | *AHR* | *STK11* | *ANXA1* | *MYOT* |
| *PRDM10* | *HSPD1* | *MFN2* | *SFTPD* | *PSMD8* |
| *RBP4* | *MB* | *RBBP4* | *TFPT* | *NFKBIE* |
| *DYNC1H1* | *BACE1* | *NOX3* | *ACAD9* | *HBE1* |
| *HPRT1* | *SIGMAR1* | *PTPN3* | *BCS1L* | *WNK1* |
| *SPARC* | *PLCG1* | *SETX* | *ACTB* | *LATS1* |
| *MAPK8IP1* | *TRAF2* | *TOR1A* | *HNF4A* | *RUVBL2* |
| *TFEB* | *PRKAA2* | *RPS6KA4* | *ALOX15* | *NUCB2* |
| *TAT* | *ABCC1* | *MRAS* | *UBE3A* | *BAG6* |
| *HDAC2* | *IGF1* | *CAMKK1* | *PTAFR* | *DDX24* |
| *VNN1* | *ERCC6* | *PPP3R1* | *LOX* | *HNRNPAB* |
| *GDF15* | *USP9X* | *CAMK1* | *ETS2* | *CASP6* |
| *SORCS2* | *TARDBP* | *BMF* | *S100A2* | *THBS1* |
| *ADSL* | *PRNP* | *PPM1L* | *ABCA1* | *ANXA6* |
| *NEFL* | *MAOB* | *SDHB* | *TSC2* | *PPA1* |
| *HPX* | *IFNG* | *ENTPD7* | *GJB2* | *ADNP* |
| *OTC* | *SLC4A11* | *IAPP* | *ITPKB* | *PSMC2* |
| *LCAT* | *FOSL1* | *ATF6* | *MSH2* | *GNAQ* |
| *RYR3* | *RETN* | *APOH* | *ABCB1* | *TGFB3* |
| *ISG15* | *RNF112* | *NDUFS7* | *PTPN2* | *PDCD10* |
| *MAP3K1* | *GSTZ1* | *ADM* | *SERPINB5* | *HAS3* |
| *BCR* | *GSTA4* | *PDX1* | *WWP2* | *MPV17* |
| *SLC25A1* | *CD36* | *INSR* | *CRAT* | *MSH6* |
| *EPHA4* | *PLCG2* | *AKR1C3* | *RECQL4* | *NTRK1* |
| *MAPKAPK5* | *BDNF* | *HEBP2* | *PPP1R15A* | *SELL* |
| *CLIC1* | *CBS* | *MTR* | *KPNA2* | *AATF* |
| *SLC7A11* | *CAMK2A* | *BLVRA* | *ALDOA* | *BARD1* |
| *TLR8* | *APOA1* | *NPM1* | *MAP3K6* | *ALPP* |
| *NCF4* | *CHKA* | *EZH2* | *VDAC1* | *NUDT5* |
| *DGKQ* | *SELE* | *KRT8* | *BCL6* | *DLD* |
| *IFNAR1* | *BMP2* | *MDM4* | *CLTC* | *MDH2* |
| *MMP8* | *LTF* | *FDXR* | *HIPK1* | *HNF1B* |
| *CDKN2B* | *ERCC2* | *MTF1* | *EDNRB* | *RNF2* |
| *TOP1* | *NDRG1* | *SHH* | *FGF1* | *SUZ12* |
| *ACO2* | *ABCD1* | *RAD51C* | *AAAS* | *AGRP* |
| *CUL3* | *JAK2* | *USP7* | *DUSP6* | *CBX4* |
| *DUOX1* | *GSTM5* | *GDNF* | *LCK* | *FBXO31* |
| *CAMK4* | *RCAN1* | *NDUFAF2* | *ODC1* | *ZAP70* |
| *CUL1* | *MUTYH* | *MAFK* | *BLVRB* | *PROS1* |
| *CYB5A* | *CHUK* | *ERCC1* | *USP28* | *HNRNPA1* |
| *IGF2BP2* | *ACO1* | *TBXA2R* | *VCP* | *CSTB* |
| *UBE2D2* | *YWHAQ* | *KNG1* | *PDIA2* | *S100A12* |
| *FAM120A* | *ALOX5* | *BCO2* | *RANBP2* | *TMBIM6* |
| *AQP4* | *ARRB2* | *RBM11* | *COL1A2* | *MME* |
| *PDGFB* | *TFRC* | *TRPC6* | *TFAP2A* | *NRG1* |
| *KCNE2* | *PCNA* | *BIRC5* | *ALDH5A1* | *HNRNPR* |
| *HAMP* | *GSTM4* | *ERN1* | *IKBKE* | *SLC16A4* |
| *BACH2* | *NTHL1* | *FTH1* | *VIP* | *ETFA* |
| *PPP5C* | *AKR1A1* | *ARG2* | *IFI16* | *CPT2* |
| *AURKA* | *GPT* | *APOA2* | *MCU* | *CGA* |
| *TLR7* | *PPARA* | *CDK2* | *PFKFB3* | *NTF3* |
| *WRN* | *REST* | *TH* | *MAP1LC3A* | *SDC4* |
| *TTPA* | *MYC* | *ADCYAP1* | *LYN* | *SERPINA3* |
| *RARA* | *HDAC1* | *GSTK1* | *PDSS2* | *PAEP* |
| *UCN* | *CDK6* | *RB1* | *SNCB* | *CARHSP1* |
| *PKP2* | *GSK3B* | *BANF1* | *CNTF* | *CETP* |
| *KCNT1* | *XRCC3* | *CTSB* | *HADHA* | *PRKCI* |
| *ATXN8OS* | *PLA2G6* | *POR* | *FOXA1* | *ELK1* |
| *MGMT* | *SFPQ* | *GHRL* | *FURIN* | *SOCS3* |
| *LIN28B* | *EEF1A1* | *DPP4* | *CD4* | *KCNJ5* |
| *SMPD1* | *ATG4D* | *RALBP1* | *TLR9* | *PAFAH2* |
| *GRM1* | *ARRB1* | *SLC23A1* | *HAX1* | *NFIB* |
| *DMPK* | *CTSD* | *TYR* | *TPO* | *AIMP2* |
| *PYCR1* | *CCS* | *TNFRSF1A* | *STC2* | *NLK* |
| *UTRN* | *SLC2A1* | *CCL5* | *RPS6KB1* | *AP2B1* |
| *FOXJ1* | *GSTM2* | *SIVA1* | *NUP98* | *TRPC5* |
| *CCR5* | *YWHAG* | *CYP1A2* | *CHRNA4* | *LCN1* |
| *SLC40A1* | *RELA* | *GJA1* | *PECAM1* | *HAS1* |
| *ITGB3* | *AKR1C1* | *RHOA* | *RDH13* | *SFRP5* |
| *CCL11* | *NFKBIA* | *NOTCH1* | *APTX* | *DAOA* |
| *MECOM* | *NR3C1* | *STK25* | *RAC2* | *DUOX2* |
| *DUSP19* | *SGK1* | *TNFSF10* | *MMACHC* | *AHCYL1* |
| *VASP* | *SERPINF1* | *STAT1* | *SUMO3* | *PIK3R1* |
| *HAO1* | *ERMP1* | *DNAJB1* | *NARF* | *CNR2* |
| *FH* | *ESR1* | *APAF1* | *NDUFAF1* | *BDKRB2* |
| *BRAF* | *DNM1L* | *RRM2B* | *EGLN1* | *CRBN* |
| *NOSIP* | *GLO1* | *CS* | *ME1* | *EIF5B* |
| *ESR2* | *E2F1* | *SMARCA4* | *NBR1* | *CHAC1* |
| *PIK3C3* | *MICB* | *ADAM17* | *MLH1* | *NXNL1* |
| *IL33* | *GPX2* | *SLC25A14* | *NPC1* | *PRKRA* |
| *CXCR1* | *UBC* | *MECP2* | *SKP2* | *ANGPT2* |
| *PRKCG* | *SREBF1* | *HBEGF* | *NSUN2* | *FEN1* |
| *CAT* | *GSTO1* | *NUP62* | *MAF* | *RNF146* |
| *SOD1* | *NR4A2* | *EIF2AK1* | *APLN* | *NPHS1* |
| *NFE2L2* | *CASP7* | *DRD2* | *ITGB1* | *PAFAH1B1* |
| *OXSR1* | *AIFM2* | *PSEN2* | *CCL27* | *FMR1* |
| *OSGIN1* | *BECN1* | *EIF1AD* | *PAX6* | *PRPF19* |
| *SOD2* | *BMP6* | *EIF4E* | *DIO1* | *TSC1* |
| *TP53* | *MMP1* | *CHEK1* | *PGK1* | *RAD52* |
| *OSGIN2* | *DCD* | *PSMB5* | *PLD2* | *PPP2R2B* |
| *GSR* | *SMAD3* | *FANCC* | *HACE1* | *RAP1A* |
| *HMOX1* | *UCP3* | *PSMA7* | *RPE* | *NUAK1* |
| *PARK7* | *PRKCZ* | *MAP4K4* | *ULK1* | *JMJD1C* |
| *MAPK8* | *PDLIM4* | *FUS* | *RNF41* | *PSMD3* |
| *SIRT1* | *PLA2G2A* | *SLC1A2* | *SERPINA1* | *RHBDF2* |
| *TXN* | *CRYAB* | *PAK2* | *BRCA2* | *S100A6* |
| *NQO1* | *SLC11A2* | *MPST* | *DDX1* | *SPAST* |
| *GPX1* | *UBB* | *NAT2* | *RPS6KA1* | *PSMD11* |
| *MAP3K5* | *NPPB* | *NPY* | *ATP7A* | *PSMD6* |
| *FOXO3* | *POLG* | *FBXO32* | *GFPT2* | *TMED4* |
| *FOXO1* | *HBB* | *NDUFAB1* | *MMP14* | *COQ10A* |
| *XDH* | *URM1* | *SLC1A3* | *DHRS2* | *WNT5A* |
| *MAPK14* | *APOB* | *SLPI* | *ANXA2* | *YY1* |
| *CASP3* | *COX5A* | *RCC2* | *NEDD4* | *F5* |
| *KEAP1* | *GSTT2* | *LIG3* | *ATXN1* | *ATP2C1* |
| *GSTM1* | *ACHE* | *HMGCR* | *HNRNPU* | *PALB2* |
| *PON1* | *CFTR* | *HSPB2* | *HNRNPK* | *HAGH* |
| *MAPK1* | *SMAD4* | *TP63* | *CBL* | *MSH3* |
| *TNF* | *EIF2AK3* | *ALDH1A1* | *PSMD1* | *ITGA3* |
| *IL6* | *GCK* | *PDIA3* | *MET* | *PPP2CA* |
| *MPO* | *PML* | *CHD6* | *FTO* | *TUBB* |
| *PRDX2* | *TJP1* | *CCL4* | *ADM2* | *NGFR* |
| *JUN* | *TNFRSF1B* | *CEBPB* | *LRP2* | *TFR2* |
| *PARP1* | *FOXM1* | *FANCG* | *FXR1* | *ITGAM* |
| *CYCS* | *EPHX1* | *GLS* | *TP73* | *RNF4* |
| *APP* | *PRKCE* | *CD40* | *TCN2* | *HNF1A* |
| *CYBA* | *PTGS1* | *RAD51* | *SHOX* | *BOLA1* |
| *TRPM2* | *CHEK2* | *MGAT3* | *TNFRSF12A* | *OSR1* |
| *SOD3* | *FOXC1* | *SDHC* | *CTCF* | *KLK3* |
| *NOS3* | *SZT2* | *GCH1* | *VNN2* | *PSMD4* |
| *GSTP1* | *TBC1D24* | *REG3A* | *CPEB2* | *TG* |
| *NOS2* | *LONP1* | *TSPO* | *PLK1* | *LGALS1* |
| *GSTT1* | *P4HB* | *TRIM25* | *DLST* | *DNAJA1* |
| *OGG1* | *TIMP1* | *NCF1* | *EWSR1* | *IMMT* |
| *CRP* | *MCL1* | *ZNF622* | *VCL* | *HSF2* |
| *G6PD* | *PIK3CA* | *NDUFA9* | *NEFH* | *ENTPD1* |
| *INS* | *SYK* | *FN1* | *VDAC2* | *NF2* |
| *PRDX3* | *OGDH* | *KRT1* | *ABCB8* | *REL* |
| *PRDX5* | *SLC25A27* | *HUWE1* | *NR1H3* | *MDH1* |
| *ABL1* | *COMT* | *NR1H2* | *OGT* | *PTPRA* |
| *BAX* | *FGF7* | *TLR3* | *NOTCH3* | *CUL2* |
| *TXNIP* | *PLAU* | *DIABLO* | *SLC18A2* | *TUFM* |
| *BCL2* | *H6PD* | *NAT1* | *MCM3* | *RPL29* |
| *PRKCD* | *NAMPT* | *RBBP7* | *KLF10* | *PTPN12* |
| *SHC1* | *PDK1* | *CREBBP* | *HNRNPM* | *MICU1* |
| *PRDX6* | *CDC37* | *F2* | *PENK* | *VSNL1* |
| *SNCA* | *APOD* | *BTRC* | *KLK1* | *STEAP1* |
| *TXNRD1* | *TRAF3* | *GSTA3* | *AMPD3* | *NADK* |
| *AKT1* | *HSPA9* | *RAN* | *TRIM32* | *HLA-G* |
| *NOX4* | *MSRB3* | *CDH1* | *PTGIS* | *HERPUD1* |
| *TXN2* | *CFH* | *DDIT4* | *SERPINI1* | *CSN1S1* |
| *UCP2* | *IRS1* | *TYRP1* | *DAO* | *ATP1A1* |
| *ALB* | *YWHAZ* | *CHRM2* | *RPE65* | *F10* |
| *HSPA4* | *MMP3* | *C11orf65* | *QDPR* | *TGIF1* |
| *CYBB* | *TFAM* | *PIK3CB* | *FANCA* | *PDGFA* |
| *APEX1* | *ECSIT* | *MC1R* | *ECE1* | *PHB2* |
| *CP* | *THBD* | *ARG1* | *MLXIPL* | *ATG9B* |
| *PRDX1* | *ISCU* | *PTPN6* | *CISD1* | *GATA4* |
| *NFKB1* | *PON3* | *UNG* | *FN3K* | *DGKK* |
| *MSRA* | *CA3* | *TIA1* | *TRIM16* | *CFL1* |
| *HSPB1* | *NFIX* | *SLC6A6* | *PLN* | *NKRF* |
| *GPX7* | *CDKN3* | *NPPA* | *LPL* | *AKR7A2* |
| *SQSTM1* | *HTRA2* | *ERCC5* | *GRPEL1* | *CRYZ* |
| *PRKD1* | *AR* | *IL17A* | *CDH2* | *FXYD2* |
| *GCLC* | *MT3* | *ITIH4* | *LDHA* | *BAG5* |
| *ADIPOQ* | *PTK2* | *SERPINA4* | *TOP2A* | *DDR2* |
| *SP1* | *CDK5* | *PIN1* | *CSK* | *FBXO7* |
| *PON2* | *IGF2BP1* | *GRB2* | *SLC12A1* | *FOXP1* |
| *TGFB1* | *HTT* | *FKBP4* | *TUBA1B* | *FZD1* |
| *GPX3* | *NDUFV1* | *HSPA6* | *METTL3* | *GPR37* |
| *MAPK10* | *PLA2G4A* | *NDUFB8* | *SPATS2L* | *GPR37L1* |
| *PRDX4* | *EGR1* | *GCG* | *TRIM33* | *MELK* |
| *MAPK3* | *TERF2* | *FGF21* | *SPG7* | *NR4A3* |
| *NOX1* | *EIF2AK2* | *RING1* | *CXCR4* | *PAWR* |
| *PINK1* | *ATXN3* | *THG1L* | *SLC25A10* | *PDE8A* |
| *FOS* |  |  |  |  |

OSRGs，Oxidative stress-responsive genes。
