# Supplementary material for: Identification of oxidative stress-responsive genes in recurrent miscarriage and their role in disease pathogenesis
Source: PLoS One. 2025 Dec 2;20(12):e0337362. doi: 10.1371/journal.pone.0337362 (PMC12671832; doi:10.1371/journal.pone.0337362)
Supplement: Attachment — Submitted filename: Response to Reviewers .docx [file pone.0337362.s003.docx]

June 9, 2025

Manuscript ID: PONE-D-25-04005

Title: Identification of oxidative stress-responsive genes in recurrent miscarriage and their role in disease pathogenesis

Dear Dr. Gary S. Stein and Reviewers,

We sincerely appreciate your valuable feedback and constructive comments on our manuscript. We have carefully addressed all points raised by the reviewers and the academic editor. The revised manuscript incorporates all suggested changes, with modifications highlighted in the “Revised Manuscript with Track Changes” file, and detailed responses are provided below. Thank you for your time and expertise in improving our work.

Journal requirements:

1. Style requirements:

We have reformatted the manuscript according to PLOS ONE templates and ensured consistent file naming.

2. Code sharing guidelines:

Not applicable (this study did not generate new code).

3. Financial disclosure:

This work was supported by the Municipal Health Commission of Nantong (No. MSZ2023057). The funders had no role in study design, data collection and analysis, decision to publish, or preparation of the manuscript.

4. Data availability statement:

All data relevant to the study are included in the article or available as supplemental information. Gene expression data are available in the GEO repository (<https://www.ncbi.nlm.nih.gov/geo/>) under accession numbers GSE22490, GSE26787, and GSE165004.

5. Supporting information captions:

Captions for supporting files are now included at the end of the manuscript, specific revisions can be found on page 72, lines 917-918.

6. Reference citations and reference list:

We carefully checked the reference citations in the text and the reference list, and removed the extra reference numbers in the main text; for more details, specific revisions can be found on page 24, line 130.

Reference list:

Reference 3: The author list format is incorrect (the abbreviation of RPL EGGo is non-standard), see page 68, line 729.

Reference 5: The page number is incorrect, see page 68, line 737.

Reference 16: The page number is incorrect, see page 69, line 769.

Reference 23: The page number is incorrect, see page 69, line 788.

Reference 28: There are multiple format errors, see page 69, lines 800-801.

Reference 35: The page number is incorrect, see page 70, line 821.

Response to Reviewers’ Comments:

Reviewer #1:

We sincerely appreciate your thorough review and valuable comments.

This study primarily conducted multi-cohort bioinformatic analyses using publicly available databases to identify OSRGs associated with RM and construct a diagnostic model. However, constrained by current resource limitations, experimental validation has not yet been performed. We plan to undertake this essential experimental validation in future work. We have explicitly acknowledged this limitation in the revised manuscript (Page 67, Lines 697-716).

Thank you for highlighting this. We have replaced all low-resolution figures with high-quality versions, with particular focus on optimizing Figures 10 and 11.

Once again, thank you for your valuable suggestions.

Reviewer #2:

We sincerely appreciate your thorough review and valuable comments.

We are particularly encouraged by your positive assessment of our study design, analytical workflow, and result interpretation. Your recognition is highly valued by our team.

Once again, thank you for your valuable suggestions.

Sincerely,

Xiaoyan Cheng.

E-mail: 1012770174@qq.com.
