# Supplementary material for: Identification of oxidative stress-responsive genes in recurrent miscarriage and their role in disease pathogenesis
Source: PLoS One. 2025 Dec 2;20(12):e0337362. doi: 10.1371/journal.pone.0337362 (PMC12671832; doi:10.1371/journal.pone.0337362)
Supplement: Attachment — Submitted filename: Response_to_Reviewers__auresp_2.docx [file pone.0337362.s004.docx]

October 13, 2025

Manuscript ID: PONE-D-25-04005

Title: Identification of oxidative stress-responsive genes in recurrent miscarriage and their role in disease pathogenesis

Dear Dr. Gary S. Stein and Reviewers,

Your comments, along with those of the other reviewers, are extremely important to our article. We greatly appreciate your insights, which have significantly contributed to enhancing the quality of our work. In this revision, we have prepared a detailed point-by-point response letter for you and the other valued reviewers. This letter acknowledges your feedback and clearly outlines the revisions we have made.

Journal requirements:

1. If the reviewer comments include a recommendation to cite specific previously published works, please review and evaluate these publications to determine whether they are relevant and should be cited. There is no requirement to cite these works unless the editor has indicated otherwise.

We sincerely appreciate your thorough review and valuable comments.

In the current round of revision, the reviewer did not suggest any specific additional citations. Our manuscript already comprehensively cites key literature and authoritative sources in the field. We are confident that the current references adequately support the scientific and methodological rigor of our study. Should any specific citation suggestions be provided in the future, we will be pleased to evaluate and incorporate them as appropriate.

Once again, thank you for your valuable suggestions.

2. Please review your reference list to ensure that it is complete and correct. If you have cited papers that have been retracted, please include the rationale for doing so in the manuscript text, or remove these references and replace them with relevant current references. Any changes to the reference list should be mentioned in the rebuttal letter that accompanies your revised manuscript. If you need to cite a retracted article, indicate the article’s retracted status in the References list and also include a citation and full reference for the retraction notice.

We sincerely appreciate your thorough review and valuable comments.

We will perform a comprehensive verification of all cited references. This process will ensure the completeness and accuracy of the reference list and confirm that all cited articles are legitimate publications, with no records of retraction or expressions of concern.

Once again, thank you for your valuable suggestions.

Response to Reviewers’ Comments:

6. Review Comments to the Author

Reviewer #4:

Minor Revisions: The abstract contains a sentence structure issue: UCN2 was positively associated with T. cells. CD8+ T cells are inversely associated with monocytes. This phrasing is confusing and requires revision for improved clarity (such as: "UCN2 demonstrated positive correlation with CD8+ T cells, which in turn showed negative correlation with monocytes"). Also, verify that gene symbols are formatted in italics consistently across the entire manuscript.

We sincerely appreciate your thorough review and valuable comments.

Regarding the suggestions for improving sentence structure clarity in the abstract and standardizing the formatting of gene symbols, we have carefully reorganized and refined the relevant content in the revised manuscript to ensure accurate and clear logical relationships.

The sentence structure issue in the abstract has been addressed: “UCN2 was positively associated with T cells” and “CD8+ T cells are inversely associated with monocytes” have been revised accordingly. Specific modifications can be found on page 3, lines 45, 46; page 46, lines 586, 588; page 53, lines 763. 764; page 54, lines 769.

We have carefully reviewed and standardized the formatting of gene symbols throughout the manuscript according to international conventions, applying italic formatting where appropriate. Italic formatting has been applied to gene symbols and related elements at the following specific locations: page 3, lines 36, 42, 44, 45; page 10, Table 2, lines 171; page 16, lines 315, 316; page 17, lines 324; page 24, lines 411, 412, 417; page 25, lines 428, 429, 433, 434, 438; page 26, lines 445, 446; page 27, lines 487, 488; page 28, lines 495; page 32, lines 547, 551; page 32-35, Table 6, lines 560; page 35-46, Table 7, lines 570; page 46, lines 585; page 48, lines 630, 631; page 70, lines 757-760.

These modifications ensure consistent and correct formatting in accordance with international scientific writing standards.

Once again, thank you for your valuable suggestions.

Although the computational analysis demonstrates technical rigor, the findings remain theoretical predictions. The authors' response to the first reviewer's concerns is inadequate. For work submitted as a "Research Article," some degree of experimental confirmation is a reasonable expectation.

I suggest implementing a major revision requirement that includes experimental corroboration of the central discoveries. The basic expectation should involve:
Quantitative RT-pCR verification of the six key genes' expression levels using a separate, small collection of clinical specimens (recurrent miscarriage patients compared to control endometrial or placental samples).
Alternatively, confirmation could be achieved through analysis of an independent publicly accessible dataset not previously utilized in the model development or validation phases.
If experimental validation cannot be provided by the authors, the manuscript would be better positioned for submission to a specialized computational biology publication or should be explicitly recharacterized as predictive research or a methodological contribution.
The fundamental analytical framework demonstrates excellent quality and deserves publication. Nevertheless, the manuscript lacks completeness in its current state. Incorporating experimental validation is essential to elevate these promising computational predictions to findings with biological and clinical significance. The authors should receive the opportunity to supply this validation prior to making a final publication decision.

We sincerely appreciate your thorough review and valuable comments.

We fully understand the recommendation to validate the six hub genes using qRT‑PCR and agree on the importance of experimental verification in enhancing the reliability of the results. Although qRT‑PCR provides sensitive and quantitative measurement of transcriptional levels, its application relies heavily on high-quality RNA and well-defined tissue sources. However, early uterine curettage tissues from patients with recurrent miscarriage are often mixtures of endometrial and decidual components, making it difficult to obtain sufficient high-quality RNA for qRT‑PCR analysis at this stage. Given these practical limitations, we plan to perform IHC analysis to validate the expression and localization of key proteins. We believe that the IHC results will strongly support our bioinformatic predictions and improve the clinical relevance of our conclusions. The corresponding revisions can be found on page 3, lines 46-49; page 13, lines 238-244; page 14, lines 245-254; page 15, lines 276-281, 287; page 47, lines 611-615, and Figure 1, Figure 12.

Once again, thank you for your valuable suggestions.

Reviewer #5（Dr. Abolfazl Akbari）:

2. Weaknesses and Key Questions for the Authors
2.1. Missing Experimental Validation:
This is the most significant limitation. The study is purely computational (bioinformatics). There is no mention of experimental validation using techniques like RT-qPCR, Western blot, or immunohistochemistry to confirm the differential expression of the identified hub genes in a separate cohort of patient samples. Without this, the findings remain hypothetical and require further verification.

Thank you for your thorough review and valuable comments.

We fully acknowledge the comment regarding the lack of experimental validation. In response, we will perform additional IHC analysis to validate key findings at the protein level, which will further corroborate the bioinformatics predictions and strengthen the persuasiveness of our study. The corresponding revisions can be found on page 3, lines 46-49; page 13, lines 238-244; page 14, lines 245-254; page 15, lines 276-281, 287; page 47, lines 611-615, and Figure 1, Figure 12.

Once again, thank you for your valuable suggestions.

2.2. Details on Results and Mechanistic Roles:
The research identifies hub genes, but it offers minimal insight into their potential mechanistic roles in the pathogenesis of RM. For instance, what is the proposed function of STK3 and its interaction with miRNAs in the context of recurrent miscarriage?
Some of the GSEA pathways mentioned (e.g., "EMT in breast tumors," "Wnt signaling in liver cancer progenitors") seem to be directly from the database output rather than being contextualized for RM. The authors should briefly explain in the full paper why these specific pathways are relevant to the disease.

We sincerely thank you for your thorough review and valuable comments.

In the revised manuscript, we will further clarify the roles of the hub genes, particularly elaborating on the interaction between STK3 and miRNAs in the pathogenesis of recurrent miscarriage. We will also supplement the biological relevance between the GSEA-enriched pathways and recurrent miscarriage to strengthen the scientific rigor and logical coherence of the findings. The corresponding revisions can be found on page 50, lines 676-684; page 52, lines 720-739; page 53, lines 740-754; page 54, lines 774-789; page 55, lines 790-802.

We once again express our gratitude for the insightful comments provided.

In addition, there are several ambiguous phrases.
The sentence "UCN2 was positively associated with T. cells. CD8+ T cells are inversely associated with monocytes" is slightly ambiguous. A reviewer would wonder if UCN2 is positively associated with both T cells and CD8+ T cells, or if this is a separate finding. The phrasing could be clearer.

We sincerely appreciate your thorough review and valuable comments.

In response to the comment regarding the need for clearer correlation statements, we will revise the manuscript to more precisely describe the correlations between UCN2 and CD8+ T cells as well as monocytes, in order to avoid any potential ambiguity. Specific modifications can be found on page 3, lines 45, 46; page 46, lines 586, 588; page 53, lines 763. 764; page 54, lines 769.

We once again express our gratitude for the insightful comments provided.

Reviewer's Recommendation
Overall, this is a promising study that lays important groundwork for future research. The article is well-structured and presents novel findings.
However, for publication in a reputable journal such as PLOS One, the authors would need to:
1. Include experimental validation of at least a few of the key hub genes (e.g., using patient samples).

We sincerely appreciate your thorough review and valuable comments.

In accordance with your comment, we will incorporate IHC analysis in the revised manuscript. These experiments will visually confirm the expression and localization of the target proteins in tissue samples, serving as crucial protein-level validation to strengthen the study's conclusions. The corresponding revisions can be found on page 3, lines 46-49; page 13, lines 238-244; page 14, lines 245-254; page 15, lines 276-281, 287; page 47, lines 611-615, and Figure 1, Figure 12.

We once again express our gratitude for the insightful comments provided.

2. Expand on the mechanistic roles of the identified genes and their potential contribution to the disease pathophysiology.

We sincerely appreciate your thorough review and valuable suggestions.

Based on your recommendations, we will further elaborate on the molecular mechanisms of the identified hub genes in the revised manuscript and systematically clarify their potential contributions to the pathogenesis of RM, so as to present a more comprehensive perspective on the functional significance of these genes. The corresponding revisions can be found on page 48, lines 634-640; page 49, lines 641-665; page 50, lines 666-690; page 51, lines 691-714; page 52, lines 715, 716.

We once again express our gratitude for the insightful comments provided.

3. This study generates excellent hypotheses, but a strong paper would need to show from correlation (bioinformatics) to causation (experimental validation).

We sincerely thank you for your thorough review and valuable suggestions.

We will incorporate IHC analysis in the revised manuscript. This experimental approach will visually demonstrate the localization and expression levels of target proteins in tissue samples, thereby providing protein-level confirmation and experimental support for the bioinformatics screening results. These additions will significantly enhance the scientific rigor and persuasiveness of our study conclusions. The corresponding revisions can be found on page 3, lines 46-49; page 13, lines 238-244; page 14, lines 245-254; page 15, lines 276-281, 287; page 47, lines 611-615, and Figure 1, Figure 12.

Once again, thank you for your valuable suggestions.

The content and language of the initial draft have undergone final optimization on the following pages and lines: page 3, lines 29-31; page 18, line 341; page 55, lines 805-814; page 56, lines 815, 817, 821, 827, 828, 831.

Sincerely,

Xiaoyan Cheng.

E-mail: 1012770174@qq.com.
